# Exploiting the Metabolic Consequences of PTEN Loss and Akt/Hexokinase 2 Hyperactivation in Prostate Cancer: A New Role for δ-Tocotrienol

**DOI:** 10.3390/ijms23095269

**Published:** 2022-05-09

**Authors:** Fabrizio Fontana, Martina Anselmi, Patrizia Limonta

**Affiliations:** Department of Pharmacological and Biomolecular Sciences, Università degli Studi di Milano, 20133 Milan, Italy; martina.anselmi@unimi.it (M.A.); patrizia.limonta@unimi.it (P.L.)

**Keywords:** prostate cancer, tocotrienols, Warburg effect, glycolysis, hexokinase 2, Akt, PTEN, metformin

## Abstract

The Warburg effect is commonly recognized as a hallmark of nearly all tumors. In prostate cancer (PCa), it has been shown to be driven by PTEN loss- and Akt hyperactivation-associated upregulation of hexokinase 2 (HK2). δ-Tocotrienol (δ-TT) is an extensively studied antitumor compound; however, its role in affecting PCa glycolysis is still unclear. Herein, we demonstrated that δ-TT inhibits glucose uptake and lactate production in PTEN-deficient LNCaP and PC3 PCa cells, by specifically decreasing HK2 expression. Notably, this was accompanied by the inhibition of the Akt pathway. Moreover, the nutraceutical could synergize with the well-known hypoglycemic agent metformin in inducing PCa cell death, highlighting the crucial role of the above metabolic phenotype in δ-TT-mediated cytotoxicity. Collectively, these results unravel novel inhibitory effects of δ-TT on glycolytic reprogramming in PCa, thus providing new perspectives into the mechanisms of its antitumor activity and into its use in combination therapy.

## 1. Introduction

Prostate cancer (PCa) is the third leading cause of cancer mortality among men in Western countries [1]. Despite advances in early detection and standard androgen-deprivation treatment, 30–40% of PCas progress towards an aggressive phenotype, often characterized by therapy resistance and poor prognosis [2,3,4]. Therefore, there is an urgent demand for the identification of novel pharmacological targets as well as of new anti-PCa agents that can complement current therapeutic strategies.

To increase macromolecule biosynthesis and sustain unlimited proliferation, most tumor cells preferentially utilize glycolysis as their major energy source, even in the presence of oxygen. This phenomenon, named the Warburg effect, is an emerging hallmark of cancer [5,6]. Hexokinase (HK), which catalyzes the conversion of glucose to glucose-6-phosphate, is the first rate-limiting enzyme in glycolysis. Four different HK isoforms have been found in mammals; however, only HK2 is responsible for the elevated aerobic glycolytic rate in tumor cells [7,8]. Overexpression of this protein has been reported in PCa, where it correlates with PTEN loss and Akt activation [9]. This evidence suggests that inhibition of the Akt/HK2-mediated Warburg effect might improve the efficacy of PCa therapy [10,11].

Tocotrienols (TTs) are vitamin E derivatives displaying potent antitumor properties [12,13,14,15]. We previously demonstrated that the δ isomer could induce mitochondrial functional and structural impairment in PCa cells [16,17]. However, the effects of δ-TT on Akt/HK2-regulated glycolysis in PTEN-deficient PCa have not been investigated yet. 

In the present study, we further dissected the molecular mechanisms underlying the antiproliferative activity of δ-TT in human PCa cells, with a focus on its ability to specifically target the Akt/HK2 signaling-associated metabolic phenotype.

## 2. Results

### 2.1. δ-TT Treatment Affects LNCaP PCa Cell Viability and Proliferation

**Figure 1:**
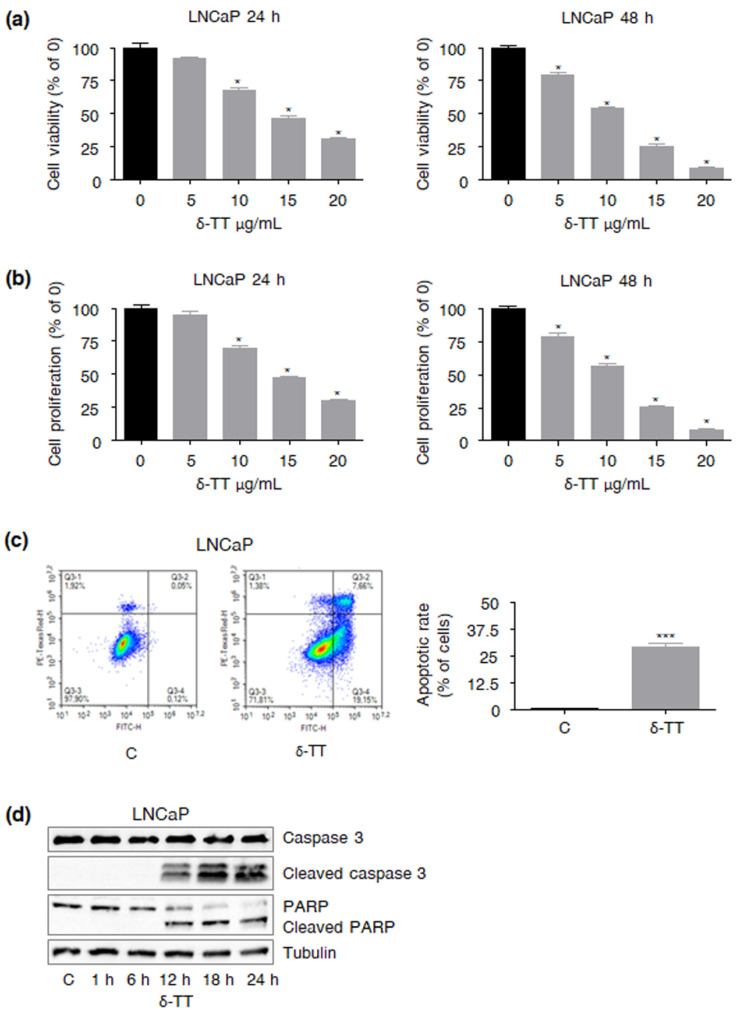
**δ-TT treatment affects LNCaP PCa cell viability and proliferation, leading to apoptosis.** (**a**) LNCaP cells were treated with δ-TT (5–20 μg/mL) for 24 and 48 h. Cell viability was then evaluated by MTT assay. Each experiment was repeated three times. Data represent mean values ± SEM and were analyzed by Dunnett’s test after one-way analysis of variance. * *p* < 0.05 vs. C, controls (vehicle). (**b**) LNCaP cells were treated with δ-TT (5–20 μg/mL) for 24 and 48 h. Cell proliferation was then evaluated by Trypan Blue exclusion assay. Each experiment was repeated three times. Data represent mean values ± SEM and were analyzed by Dunnett’s test after one-way analysis of variance. * *p* < 0.05 vs. C, controls (vehicle). (**c**) LNCaP cells were treated with δ-TT (15 μg/mL, 24 h); apoptotic rate was then evaluated by cytofluorimetric analysis after staining with eBioscience™ Annexin V-FITC Apoptosis Detection Kit (according to the manufacturer’s protocol). Each experiment was repeated three times. Data represent mean values ± SEM and were analyzed by *t*-test. *** *p* < 0.001 vs. C, controls (vehicle). (**d**) After δ-TT treatment (15 μg/mL, 1–24 h), Western blot analysis was performed to investigate the expression levels of cleaved caspase 3 and PARP. Tubulin expression was evaluated as a loading control. One representative of three experiments performed is shown.

We have previously reported that δ-TT can inhibit castration-resistant PCa cell growth [16]. Here, we showed that it can also dose- and time-dependently reduce LNCaP androgen-sensitive PCa cell viability and proliferation (δ-TT 5–20 μg/mL, 24–48 h), as evidenced by MTT and Trypan blue exclusion assays (Figure 1a,b). In particular, its IC50 at 24 h was found to be 3.54 × 10^−5^ M; thus, δ-TT 15 μg/mL, a cytotoxic dose close to this value, was selected as an appropriate concentration for further experiments.

### 2.2. δ-TT Induces Apoptosis in LNCaP PCa Cells

The specific molecular mechanisms by which δ-TT (15 μg/mL) could induce LNCaP cell death were next investigated. Interestingly, the compound was found to trigger apoptosis, with mortality rates being around 30% (24 h, Figure 1c). This hypothesis was also confirmed by the parallel cleavage of caspase 3 and PARP (24 h, Figure 1d). Overall, these results demonstrated the involvement of the apoptotic pathway in the anti-PCa activity of the compound, which appears to be effective regardless of the specific tumor stage.

### 2.3. δ-TT Cytotoxicity Correlates with Suppression of the HK2-Driven Warburg Effect in LNCaP PCa Cells

**Figure 2:**
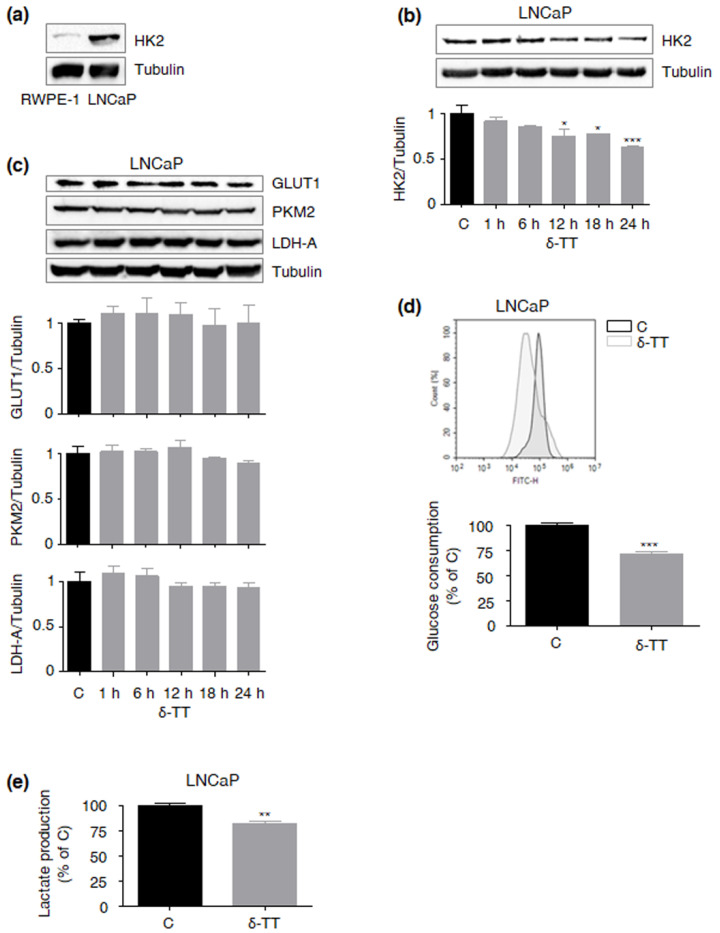
**δ-TT cytotoxicity correlates with the suppression of the HK2-driven Warburg effect in LNCaP PCa cells.** (**a**) HK2 expression was evaluated in RWPE-1 and LNCaP cells by Western blot analysis. Tubulin expression was evaluated as a loading control. One representative of three experiments performed is shown. (**b**) After δ-TT treatment (15 μg/mL, 1–24 h), Western blot analysis was performed to investigate the expression levels of HK2 in LNCaP cells. Tubulin expression was evaluated as a loading control. Data represent mean values ± SEM and were analyzed by Dunnett’s test after one-way analysis of variance. * *p* < 0.05 vs. C, controls (vehicle); *** *p* < 0.001 vs. C, controls (vehicle). (**c**) After δ-TT treatment (15 μg/mL, 1–24 h), Western blot analysis was performed to investigate the expression levels of GLUT1, PKM2 and LDH-A in LNCaP cells. Tubulin expression was evaluated as a loading control. Data represent mean values ± SEM and were analyzed by Dunnett’s test after one-way analysis of variance. No significant changes in protein expression were observed. (**d**) LNCaP cells were treated with δ-TT (15 μg/mL, 18 h); glucose consumption was then evaluated by cytofluorimetric analysis after staining with 2-(N-(7-Nitrobenz-2-oxa-1,3-diazol-4-yl)Amino)-2-Deoxyglucose (2-NBDG) 100 μM for 30 min. Each experiment was repeated three times. Data represent mean values ± SEM and were analyzed by *t*-test. *** *p* < 0.001 vs. C, controls (vehicle). (**e**) LNCaP cells were treated with δ-TT (15 μg/mL, 18 h); lactate production was then evaluated by colorimetric assay. Each experiment was repeated three times. Data represent mean values ± SEM and were analyzed by *t*-test. ** *p* < 0.01 vs. C, controls (vehicle).

The LNCaP cell line has been recently proposed as a model of HK2-overexpressing PCa [9]. After confirming that these cells display this specific molecular signature (compared with RWPE-1 normal prostate epithelial cells) (Figure 2a), we investigated whether δ-TT could target the associated Warburg effect. Figure 2b shows that in this cell line, the compound (15 μg/mL, 24 h) can induce a time-dependent decrease in HK2 protein levels, without affecting the expression of other key glycolytic proteins, such as glucose transporter 1 (GLUT1), pyruvate kinase M2 (PKM2) and lactate dehydrogenase A (LDH-A) (Figure 2c). As expected, HK2 downregulation was followed by inhibition (at 18 h) of glucose consumption (Figure 2d) and lactate production (Figure 2e). Collectively, these data highlight the ability of δ-TT to severely alter HK2-regulated aerobic glycolysis in LNCaP cells.

### 2.4. δ-TT Downregulates HK2 via Specific Inhibition of Akt in PTEN-Null PCa Cells

**Figure 3:**
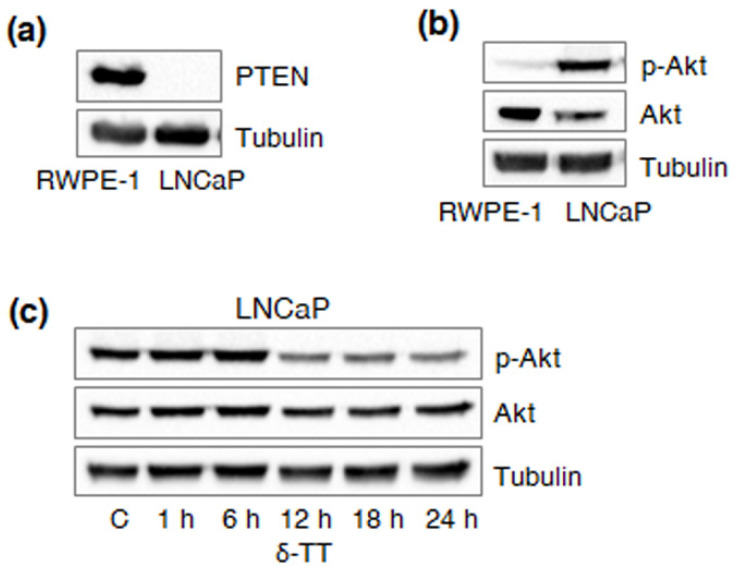
**δ-TT downregulates HK2 in LNCaP PCa cells via inhibition of Akt.** (**a**) PTEN expression was evaluated in RWPE-1 and LNCaP cells by Western blot analysis. Tubulin expression was evaluated as a loading control. One representative of three experiments performed is shown. (**b**) Akt hyperactivation was evaluated in RWPE-1 and LNCaP cells by Western blot analysis. Tubulin expression was evaluated as a loading control. One representative of three experiments performed is shown. (**c**) After δ-TT treatment (15 μg/mL, 1–24 h), Western blot analysis was performed to investigate the expression levels of p-Akt in LNCaP cells. Tubulin expression was evaluated as a loading control. One representative of three experiments performed is shown.

**Figure 4:**
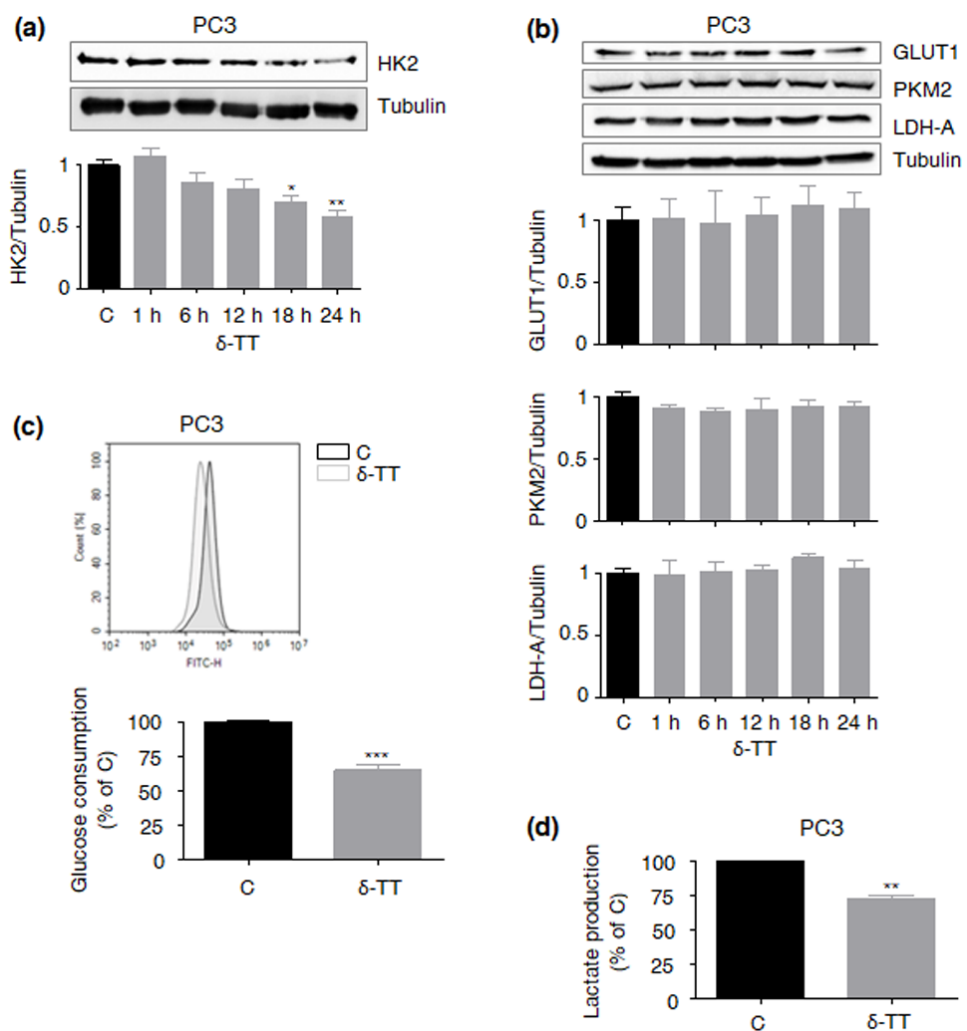
**δ-TT targets the Akt/HK2-driven Warburg effect also in PC3 PCa cells.** (**a**) After δ-TT treatment (15 μg/mL, 1–24 h), Western blot analysis was performed to investigate the expression levels of HK2 in PC3 cells. Tubulin expression was evaluated as a loading control. Data represent mean values ± SEM and were analyzed by Dunnett’s test after one-way analysis of variance. * *p* < 0.05 vs. C, controls (vehicle); ** *p* < 0.01 vs. C, controls (vehicle). (**b**) After δ-TT treatment (15 μg/mL, 1–24 h), Western blot analysis was performed to investigate the expression levels of GLUT1, PKM2 and LDH-A in PC3 cells. Tubulin expression was evaluated as a loading control. Data represent mean values ± SEM and were analyzed by Dunnett’s test after one-way analysis of variance. No significant changes in protein expression were observed. (**c**) PC3 cells were treated with δ-TT (15 μg/mL, 18 h); glucose consumption was then evaluated by cytofluorimetric analysis after staining with 2-(N-(7-Nitrobenz-2-oxa-1,3-diazol-4-yl)Amino)-2-Deoxyglucose (2-NBDG) 100 μM for 30 min. Each experiment was repeated three times. Data represent mean values ± SEM and were analyzed by *t*-test. *** *p* < 0.001 vs. C, controls (vehicle). (**d**) PC3 cells were treated with δ-TT (15 μg/mL, 18 h); lactate production was then evaluated by colorimetric assay. Each experiment was repeated three times. Data represent mean values ± SEM and were analyzed by *t*-test. ** *p* < 0.01 vs. C, controls (vehicle).

In PCa cells, including LNCaP cell line, HK2 upregulation has been associated with PTEN deficiency and consequent Akt hyperactivation [9]. After validating our model in terms of PTEN and Akt expression (Figure 3a,b), we demonstrated that δ-TT (15 μg/mL, 24 h) can time-dependently inhibit the latter (Figure 3c). In particular, upon δ-TT treatment, decreased HK2 expression coincided with reduced p-Akt levels, suggesting that the compound can downregulate this enzyme by inactivating the Akt signaling. To test this hypothesis, we also examined the anti-glycolytic activity of δ-TT (15 μg/mL, 24 h) in PC3 castration-resistant PCa cells, known to be PTEN-deficient and to undergo Akt dephosphorylation-related apoptosis after exposure to the nutraceutical [9,11,17]. As expected, specific downregulation of HK2, followed by reduced glucose uptake and lactate release (at 18 h), was observed in this cell line (Figure 4a–d). Contrarily, no significant changes in the glycolytic rate were found in DU145 androgen-unresponsive PCa cells (data not shown), previously reported to be PTEN-wild type and Akt/HK2-null [9,17]. Altogether, these findings emphasize the vulnerability of PCas characterized by a glucose-dependent metabolic phenotype to δ-TT anti-tumor effects. 

### 2.5. δ-TT Synergizes with Metformin in Reducing PTEN-Null and Akt/HK2-Overexpressing PCa Cell Viability

**Figure 5:**
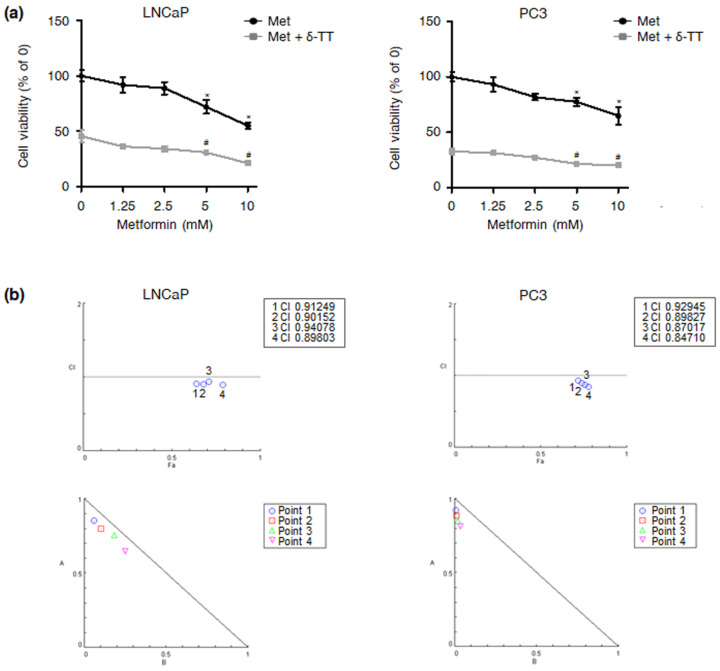
**δ-TT synergizes with metformin in reducing PTEN-null and Akt/HK2-overexpressing PCa cell viability.** (**a**) LNCaP and PC3 cells were treated with metformin (1.25, 2.5, 5, 10 mM), alone or in combination with δ-TT (15 μg/mL) for 24 h. Cell viability was then evaluated by MTT assay. Each experiment was repeated three times. Data represent mean values ± SEM and were analyzed by Dunnett’s test after one-way analysis of variance. * *p* < 0.05 vs. C, controls (vehicle). # *p* < 0.05 vs. δ-TT-treated cells. (**b**) Data from Figure 5a were converted to Fraction Affected (FA) and plotted against Combination Index (CI). Straight line on the graph designates a CI equal to 1. Combination Index interpretation was as follows: CI value of 1 indicates additivity; CI < 1 indicates synergism; and CI > 1 indicates antagonism. Points 1, 2, 3 and 4 are the combination of δ-TT 15 μg/mL and metformin 1.25, 2.5, 5 or 10 mM, respectively.

Based on these observations, we finally verified if δ-TT could enhance the cytotoxicity of the hypoglycemic drug metformin, currently under study for PCa management [18,19,20]. As shown in Figure 5a,b, the nutraceutical was able to synergize with the anti-diabetic therapeutic in inducing LNCaP and PC3 cell death, demonstrating great potential in both mono- and combination therapy when used to treat PTEN-null and Akt/HK2-overexpressing PCa.

## 3. Discussion

In recent years, cancer metabolic reprogramming has gained increasing attention due to its immense potential as a crucial pharmacological target. In particular, metabolic enzymes might represent vital therapeutic hotspots, based on their role as key regulators of tumor cell proliferation [21]. A large body of evidence suggests that HK2 is fundamental in modulating cancer glycolysis, and several compounds have shown promise in suppressing cancer growth by specifically inhibiting this protein [22,23]. In PCa, HK2 upregulation correlates with PTEN deficiency and subsequent Akt hyperactivation [9], suggesting that targeting the Akt/HK2 axis could represent an effective antitumor strategy [10,11]. 

In the present study, we investigated whether δ-TT, a well-known antitumor nutraceutical, was able to specifically target the Akt/HK2-driven Warburg effect in PCa cells.

First, we demonstrated that δ-TT can exert significant antitumor effects, not only in androgen-independent but also in androgen-sensitive PCa cells, by decreasing both cell viability and proliferation regardless of the tumor stage. Specifically, we observed that the compound can trigger apoptosis, paralleled by caspase 3 and PARP cleavage. This is in agreement with recent findings evidencing the proapoptotic effects elicited by TTs in various malignancies, such as skin, pancreatic, lung, liver, gastric, colon, ovarian and breast cancer [12,13,24,25,26,27].

Next, we found that δ-TT treatment leads to a specific decrease in HK2 protein levels in PTEN-null PCa cells, without altering the expression of other key glycolytic enzymes, including GLUT1, PKM2 and LDH-A; as expected, these changes were followed by a significant reduction in glucose consumption and lactate production. Interestingly, HK2 downregulation coincided with the suppression of the Akt pathway, highlighting the ability of δ-TT to selectively modulate the Akt/HK2 signaling in PTEN-deficient PCa. These data are consistent with previous reports describing the crucial role of the Akt/HK2 cascade in the cytotoxicity induced by different natural products, such as daphnetin, deguelin, jolkinolde B, licochalcone A, limonin, piperlongumine, sinomenine and xanthohumol, in several malignancies [28,29,30,31,32,33,34,35]. Regarding TTs, the γ isoform has been recently shown to suppress the Warburg effect in breast cancer [36]; our results demonstrate for the first time that the δ component is also endowed with anti-glycolytic properties, being able to selectively target the aberrant glucose metabolism of PCa.

A large body of evidence suggests that direct targeting of specific metabolic traits can successfully lead to cancer eradication [37,38]. In this regard, metformin, a well-established anti-diabetic molecule, has recently emerged as a promising anti-PCa agent [18,19,20]. Based on these observations, we conducted a combination study with this drug and δ-TT, evidencing that the phytochemical could sensitize PCa cells characterized by the Warburg effect to hypoglycemic therapy. To our knowledge, this is the first study highlighting the synergism between δ-TT and metformin in PCa.

In conclusion, these results provide a deeper understanding of the anticancer properties of δ-TT, demonstrating that it exerts potent antitumor activity in PTEN-null and Akt/HK2-overexpressing PCa cells by targeting their specific metabolic features and that it might serve as a promising therapeutic strategy when given in combination with anti-glycolytic agents.

## 4. Materials and Methods

### 4.1. Chemicals

δ-TT was purified from a commercial extract of Annatto seeds (*Bixa orellana* L.) (kindly provided by American River Nutrition Inc., Hadley, MA, USA), as previously described [25]. 

The primary antibodies caspase 3 (9656), cleaved caspase 3 (9664), PARP (9542), PTEN (9188), HK2 (2867), Akt (2938) and p-Akt (9271), GLUT1 (12,939) and PKM2 (4053) were from Cell Signaling Technology Inc. (Danvers, MA, USA), LDH-A (sc-13,7243) was from Santa Cruz Biotechnology Inc. (Santa Cruz, CA, USA), and α-tubulin (T6199) was from Sigma-Aldrich (Milano, Italy). All of the antibodies were used at the concentration 1:1000.

Horseradish-peroxidase-conjugated secondary antibody and enhanced chemiluminescence reagents were from Cyanagen (Bologna, Italy). 

### 4.2. Cell Lines and Cell Culture

PTEN-deficient LNCaP and PC3 PCa cells and PTEN-wild type DU145 PCa cells were from American Type Culture Collection (ATCC, Manassas, VA, USA), and they were cultured in RPMI medium supplemented with 7.5% FBS, glutamine and antibiotics, in a humidified atmosphere of 5% CO_2_/95% air at 37 °C. Normal prostate epithelial RWPE-1 cell line was also from ATCC and was grown in keratinocyte-SFM medium supplemented with bovine pituitary extracts and EGF (2.5 μM) (Thermo Fisher Scientific, Waltham, MA, USA). Original stocks of cells were stored frozen in liquid nitrogen; after resuscitation, cells were kept in culture for no more than 10–12 weeks. Cells were detached through trypsin-EDTA solution and passaged once/week.

### 4.3. MTT Viability Assay

Cells were seeded at a density of 3 × 10^4^ cells/well in 24-well plates for 48 h and then exposed to the specific compounds. The medium was then changed with MTT solution (0.5 mg/mL) in RPMI without phenol red and FBS; cells were incubated at 37 °C for 30 min, and violet precipitate was dissolved with isopropanol. Absorbance at 550 nm was measured through an EnSpire Multimode Plate reader (PerkinElmer, Milano, Italy).

### 4.4. Trypan Blue Exclusion Assay

Cells were plated (1.5 × 10^5^ cells/dish) in 6 cm dishes. After 48 h, cells were treated with δ-TT (5–20 μg/mL, 24 h). Adherent (viable) and floating (dead) cells were harvested, stained with Trypan blue 0.4% (1:1 *v*/*v*) and counted by Luna automated cell counter (Logos Biosystems, Annandale, VA, USA).

### 4.5. Annexin V/PI Apoptosis Assay

Cells were plated (1.5 × 10^5^ cells/dish) in 6 cm dishes. After 48 h, cells were treated with δ-TT (15 μg/mL, 24 h). Adherent (viable) and floating (dead) cells were harvested, washed in PBS and incubated with Annexin V and PI, using the eBioscience™ Annexin V-FITC Apoptosis Detection Kit. The flow cytometry analyses were performed with a Novocyte3000 instrument (ACEA Biosciences, San Diego, CA, USA). Data were analyzed with Novoexpress software. 

### 4.6. Measurement of Glucose Consumption

Cells were plated (1.5 × 10^5^ cells/dish) in 6 cm dishes. After 48 h, cells were treated with δ-TT (15 μg/mL, 18 h). Adherent (viable) and floating (dead) cells were harvested, washed in PBS and incubated with 2-(N-(7-Nitrobenz-2-oxa-1,3-diazol-4-yl)Amino)-2-Deoxyglucose (Thermo Fisher Scientific) 100 μM for 30 min. The flow cytometry analyses were performed with a Novocyte3000 instrument. Data were analyzed with Novoexpress software.

### 4.7. Measurement of Lactate Production

Lactate production was measured by using a lactate assay kit (Sigma-Aldrich). An EnSpire Multimode Plate reader (PerkinElmer, Milano, Italy) was used to quantify absorbance at 570 nm.

### 4.8. Western Blot Analysis

Cells were seeded at 5 × 10^5^ cells/dish in 10 cm dishes. After each treatment, cells were lysed in RIPA buffer; protein preparations (25 μg) were resolved on SDS-PAGE and transferred to nitrocellulose membranes. Membranes were incubated with the specific primary antibodies. Detection was performed using horseradish peroxidase-conjugated secondary antibodies and enhanced chemiluminescence (Westar Etac Ultra 2.0, XLS075,0100, Cyanagen). α-Tubulin was utilized as a loading control.

### 4.9. Isobologram Analysis

Cells were treated for 24 h using four different concentrations of metformin and δ-TT 15 μg/mL. Viable cells were quantitated by MTT assay as described above, and CalcuSyn software was used to generate an isobologram.

### 4.10. Statistical Analysis

Statistical analysis was performed with a statistic package (GraphPad Prism5, GraphPad Software San Diego, CA, USA). Data are represented as the mean ± SEM of three or four independent experiments. Differences between groups were assessed by *t*-test or one-way analysis of variance (ANOVA) followed by Dunnett’s test. A *p* value < 0.05 was considered statistically significant.

## Data Availability

Not applicable.
